# Differentiation of Bone Marrow Mesenchymal Stem Cells to Cardiomyocyte-Like Cells Is Regulated by the Combined Low Dose Treatment of Transforming Growth Factor-*β*1 and 5-Azacytidine

**DOI:** 10.1155/2016/3816256

**Published:** 2015-11-30

**Authors:** Shutian Shi, Xingxin Wu, Xiao Wang, Wen Hao, Huangtai Miao, Lei Zhen, Shaoping Nie

**Affiliations:** ^1^Emergency Center, Anzhen Hospital, Capital Medical University, Beijing 100029, China; ^2^Beijing Institute of Heart Lung and Blood Vessel Diseases, Beijing 100029, China

## Abstract

Bone marrow mesenchymal stem cells (BMMSCs) are used in cardiac tissue engineering for the regeneration of diseased hearts. We examined the differentiation of rat BMMSCs into cardiomyocyte-like cells when induced with a combined low dose treatment of transforming growth factor-*β*1 (TGF-*β*1) and 5-azacytidine (5-AZA). Results showed that cell proliferation in the combined low dose treatment group of TGF-*β*1 and 5-AZA was increased compared with the TGF-*β*1 group or the 5-AZA group. The cell apoptosis was relieved by combined TGF-*β*1 and 5-AZA treatment compared to 5-AZA treatment alone. The number of cells positive for myosin heavy chain, connexin-43, *α*-actin, and troponin I in the combined treatment group was higher than those observed in the TGF-*β*1 group or the 5-AZA group. Moreover, the combined low dose treatment group of TGF-*β*1 and 5-AZA reveals the strongest expression of troponin I, *α*-actin, and phosphorylated extracellular signal-regulated protein kinases 1 and 2 (p-ErK1/2) among the treatment groups. These results suggest that the combined low dose treatment of TGF-*β*1 and 5-AZA can improve the differentiation potential of rat BMMSCs into cardiomyocyte-like cells and alleviate cell damage effects *in vitro*. The mechanism that is involved in influencing differentiation may be associated with p-ErK1/2.

## 1. Introduction

Ischemic heart disease is a leading cause of morbidity and mortality in developed countries and is one of the major contributors of disease burden in developing countries [1, 2]. The current treatment techniques that include medications, interventional procedures, and surgery can only open occluded coronary arteries or provide symptomatic relief without addressing the major issue of the cumulative loss of functioning cardiomyocytes [3–5]. Cellular therapy offers another option in the treatment of ischemic heart diseases. Bone marrow-derived mesenchymal stem cells (BMMSCs) have been considered one of the best options for cellular therapy because of their immunological naivety, self-renewing capacity, and multipotent differentiation property [6].

Experimental evidence clearly demonstrated that BMMSCs may differentiate into cardiomyocytes* in vitro* and* in vivo* and may be home to areas of ischemic injury following the administration of BMMSCs, to provide functional recovery [7]. Many studies have shown that 5-azacytidine (5-AZA) can induce BMMSCs to differentiate into cardiomyocytes [8–10]. It is well known that transforming growth factor-*β*1 (TGF-*β*1) can play a similar role in the cardiac differentiation process of BMMSCs [10–12]. However, 5-AZA can affect cell survival after induction because of its obvious toxicity as an antineoplastic agent [10], while TGF-*β*1 reveals very low efficiency [11]. One of the main challenges of cell therapy is to identify an induction method with a high differentiation rate and low cytotoxicity.

In the present study, we investigated the effect of combined low dose treatment of TGF-*β*1 and 5-AZA, on cardiomyogenic differentiation of BMMSCs. Our results demonstrate that the combination of low doses of TGF-*β*1 and 5-AZA can significantly improve the cardiac differentiation rate and exhibit lower cell damage effects. In order to analyze the mechanisms involved in differentiation, we investigated the protein levels of phosphorylated extracellular signal-regulated kinases 1 and 2 (p-ErK1/2) following induction.

## 2. Materials and Methods

### 2.1. Isolation and Culture of BMMSCs

BMMSCs were obtained from the femur and the tibial bone marrow of 4-week-old Sprague-Dawley (SD) rats (Vital River Laboratory Animal Technology Co. Ltd., Beijing, China) by density-gradient centrifugation, as previously described [13]. All the animal experimental procedures and protocols used in the present study conform to the standards for laboratory animals established by China (GB14925-2001), and all the research was approved by the Committee on Ethics of Animal Experiments of Capital Medical University. The cells were grown in complete low glucose DMEM/F-12 medium (L-DMEM, Thermo Fisher Scientific Inc., MA, USA) supplemented with 10% fetal bovine serum (Thermo Fisher Scientific Inc., MA, USA) and antibiotics (100 U/mL penicillin + 100 *μ*g/mL streptomycin) at 37°C in a humidified atmosphere of 5% CO_2_ and 21% O_2_. The culture medium was replenished every 3 days. Upon reaching 80%–90% confluence, the cells were detached with 0.25% trypsin and passaged further in a 1 : 2 ratio. For all the subsequent experiments, cells from the third passage were used.

### 2.2. Identification of BMMSCs

The BMMSCs were harvested and resuspended at 1 × 10^6^/mL. The cells were stained with labeled antibody and incubated at 4°C for 30 min in the dark. The following anti-rat antibodies were used: CD19-PE, CD29-PE, CD34-FITC, CD44-FITC, CD45-FITC, and CD90-PE. All the antibodies were purchased from BD Biosciences (BD Biosciences, CA, USA). After washing twice with phosphate-buffered solution (PBS), the positive rates of surface antigen were detected with flow cytometry.

### 2.3. Induction of BMMSCs

The BMMSCs were divided into 4 groups as follows: (1) control group: the BMMSCs maintained only in basal media were used as the assay control; (2) TGF-*β*1 group: the BMMSCs were exposed to 10 ng/mL TGF-*β*1 (PeproTech, NJ, USA) for 14 days [12]; (3) 5-AZA group: the BMMSCs were exposed to 10 *μ*mol/L of 5-AZA (Sigma-Aldrich Co. LLC, MO, USA) for 24 h and maintained in basal media alone for the next 28 days [14]; (4) combined low dose treatment group of TGF-*β*1 and 5-AZA: the BMMSCs were treated with 5 *μ*mol/L 5-AZA and 5 ng/mL TGF-*β*1 for 24 h, then washed twice with PBS, and maintained with 5 ng/mL TGF-*β*1 alone for up to 14 days. For analysis of the mechanisms involved in the differentiation, a specific inhibitor of ErK, U0126 (10 *μ*mol/L, Promega Corporation, Beijing, China), was added to the medium until the cells were collected.

### 2.4. MTS Cell Proliferation Assay

The MTS assays were performed to provide a preliminary assessment of toxicity of the inducing agents. The proliferation of the cells in each group was detected using the MTS solution cell proliferation assay kits (Promega Corporation, WI, USA). The MTS assays were performed at days 1, 3, 5, and 7. The absorbance at 490 nm was read using a SpectraMax M2 microplate reader (Molecular Devices, LLC, CA, USA), and the final absorption (A) values were the values in each group minus the background absorbance values. The cell proliferation curve was then established.

### 2.5. Annexin V Binding Apoptosis Assay

Following 24 h incubation, BMMSCs in the three treatment groups and the control group were digested with trypsin and washed two times with cold PBS. Apoptosis was detected by flow cytometry using an FITC annexin V apoptosis detection kit (BD Pharmingen, Inc., San Diego, CA, USA). Cells were resuspended in binding buffer, stained with FITC annexin V and propidium iodide for 15 minutes at room temperature in the dark. Add 400 *μ*L of 1X binding buffer to each tube. Finally the cells were subjected to flow cytometry analysis for apoptosis within 1 h. Fluorescence data were collected using an EPICS XL Flow Cytometer (Beckman Coulter, Inc., San Diego, CA, USA) and analyzed using the EXPO32 ADC software (Beckman Coulter).

### 2.6. Immunofluorescence Staining of Cardiac-Specific Proteins

To identify whether BMMSCs are induced to differentiate into cardiomyocytes by a low dose combination of TGF-*β*1 and 5-AZA, we performed immunofluorescence staining of cardiac-specific proteins. Cells of each group were fixed with 4% paraformaldehyde at 4°C for 30 min. After the cells were blocked with fetal calf serum for 1 h at room temperature, the cells were incubated with 0.5% TritonX-100 for 30 min and washed twice with PBS. The cells were incubated with each of the antibodies for anticardiac troponin I (cTnI), *α*-actin, myosin heavy chain (MHC), and connexin-43 (CX-43), at 4°C for 24 h. All the antibodies were purchased from abcam (abcam, Cambridge, UK) and the dilution rate was 1 : 250. After a second double wash with PBS, the cells were labeled with the respective secondary antibody for 30 min at room temperature in the dark. The images were obtained using a Nikon Eclipse 600 microscope equipped with a DS-Ri1 camera (Nikon Instruments Inc., Tokyo, Japan). The images were processed using the NIS-Elements AR analysis software (Nikon Instruments).

### 2.7. Flow Cytometry Analysis of the Differentiation Rate

We performed a flow cytometry analysis to compare the differentiation rate of BMMSCs to cardiomyocytes in different groups. The cells were first trypsinized, washed twice with PBS, and aliquoted into 1 × 10^6^ cells/100 *μ*L in Fluorescence Activated Cell Sorting (FACS) tubes. 0.5 mL of cold flow cytometry fixation buffer (Shanghai Yeasen Biotechnology Co., Ltd, Shanghai, China) was added and the cells were incubated at room temperature for 10 min. The cells were washed twice with PBS and then resuspended in 1.5 mL of cold permeabilization wash buffer (Yeasen Biotech Co., Ltd, Shanghai, China) and vortexed. The cells were then incubated at room temperature for 10 min. The cells were centrifuged for 5 min at 1000 ×g. The supernatant was removed, discarded, and then incubated with cTnI antibody for 1 h. After washing twice, the cells were stained with a FITC-conjugated secondary antibody (BD Biosciences) for 30 min. For the negative controls, the cells were incubated only with a FITC-conjugated secondary antibody. Finally, cell suspensions were fixed in ice-cold 2% paraformaldehyde for flow cytometric analysis. Fluorescence data were collected using an EPICS XL Flow Cytometer (Beckman Coulter) and analyzed using the EXPO32 ADC software (Beckman Coulter).

### 2.8. Western Blot Analysis

The protein expression of cTnI, *α*-actin, p-ErK1/2, total-ErK (t-ErK), and octamer-binding transcription factor 4 (OCT4) from the 4 groups of cells were determined by western blot analysis using antibodies against cTnI, *α*-actin, p-ErK1/2, t-ErK, and OCT4, as described previously. All the antibodies were purchased from abcam.

The levels of cTnI, *α*-actin, and OCT4 protein were detected at the end of induction, and the levels of p-ErK1/2 and t-ErK were detected 7 days after induction. We measured the total protein using the bicinchoninic acid protein assay kit (Beyotime Institute of Biotechnology, Jiangsu, China). GAPDH served as the loading control on the same membrane. We visualized the results of the western blot analysis with Fluor-S-Imager using Quantity One V4.6 software (Bio-Rad Laboratories, Inc., CA, USA).

### 2.9. Statistical Analysis

All the data were presented as mean ± SEM. SPSS version 19.0 was used for data analysis. Multiple group comparisons were performed by one-way ANOVA. Statistical significance was accepted at *P* < 0.05.

## 3. Results

### 3.1. Cellular Characterization

**Figure 1:**
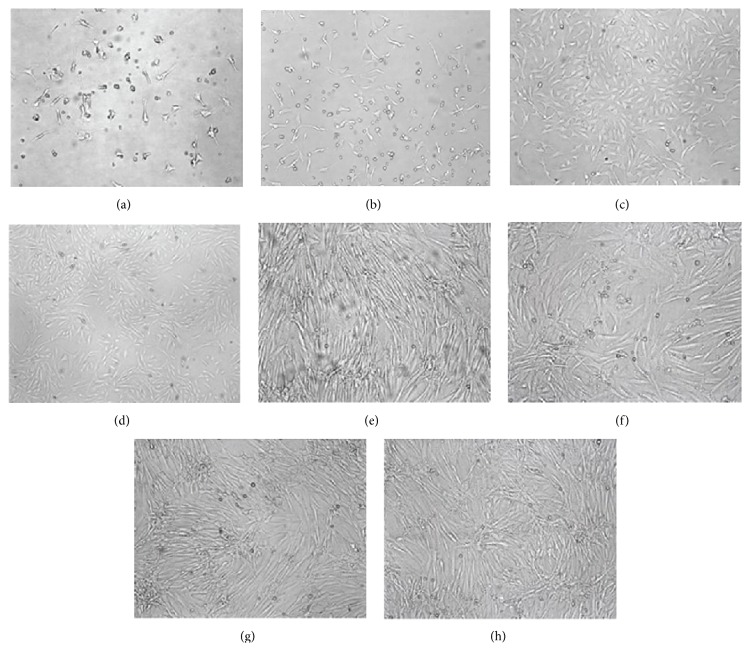
Cellular characterization. (a) Primary culture 24 h; (b) primary culture 3 d; (c) primary culture 9 d; (d) passage 3; (e) control group; (f) TGF-*β*1 group; (g) 5-AZA group; (h) TGF-*β*1 + 5-AZA group.

Cells were observed under the light microscope Leica CTR 4000 (Leica Microsystems GmbH, Wetzlar, Germany) at different time points, and images were taken using the Leica Application Suite Version 4.1.0 (Figures 1(a), 1(b), 1(c), and 1(d)). Primary BMMSCs were round and floated in the culture medium. After 24 h of primary culture, there were only a small number of cells that adhered to the plastic surface. Three days later, the adhered cells developed a circular morphology. Following several days in culture, the cells developed a typical spindle-shaped morphology. These cells obtained confluence, of approximately 80% to 90%, within 8 to 10 days of cultivation. When subcultured, the cells ended up either as polygonal or as long, spindle-shaped cells. These cells were confirmed as mesenchymal stem cells (MSCs) as they stained positive for CD29 (90.26 ± 1.41%), CD44 (93.44 ± 3.65%), and CD90 (98.82 ± 4.03%), while they stained negative for CD19 (3.61 ± 0.37%), CD34 (2.92 ± 0.29%), and CD45 (1.87 ± 0.44%). The final cell morphology of the 3 groups showed no difference following treatment (Figures 1(e), 1(f), 1(g), and 1(h)).

### 3.2. Cell Proliferation and Apoptosis

**Figure 2:**
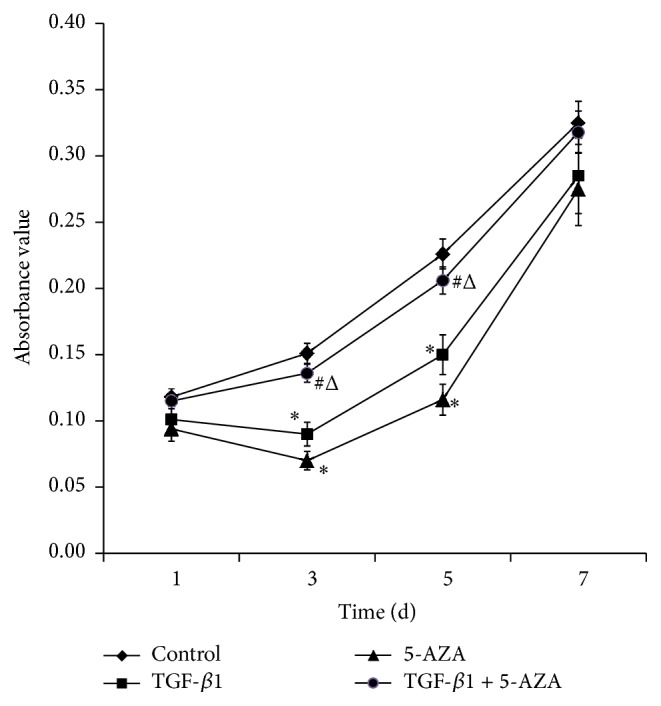
Cells proliferation in different groups. The *x*-axis represents time, while the *y*-axis represents the absorption values. Results are expressed as means ± SEM. ^*∗*^ *P* < 0.05 versus control group; ^#^ *P* < 0.05 versus TGF-*β*1 group; ^Δ^ *P* < 0.05 versus 5-AZA group (*n* = 6).

The MTS assay demonstrated that cell proliferation in the TGF-*β*1 group as well as in the 5-AZA group was slightly inhibited on the third day followed by a gradual increase. The cells in the control group and the combined treatment group, TGF-*β*1 + 5-AZA, showed only a slight increasing trend. The cell proliferation in the TGF-*β*1 group as well as the 5-AZA group decreased compared with the control group on the 3rd and the 5th days, while the cell proliferation in the combined treatment group, TGF-*β*1 + 5-AZA, demonstrated no significant change. In addition, there was no significant difference in cell proliferation on the 7th day (Figure 2).

**Figure 3:**
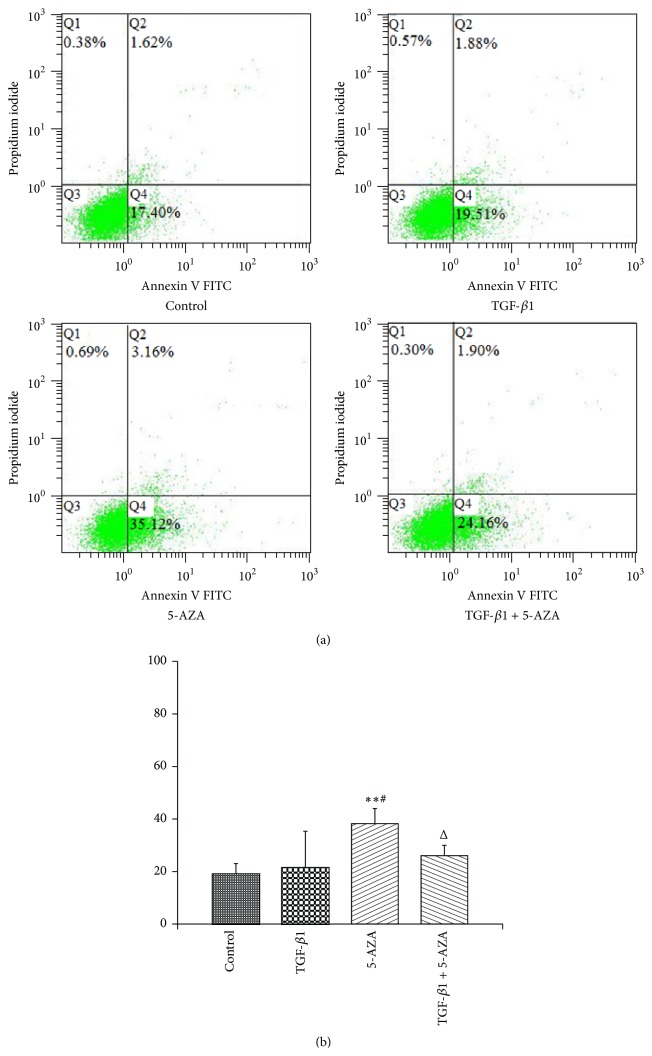
Cells apoptosis in different groups. Results are expressed as the means ± SEM. ^*∗∗*^ *P* < 0.01 versus control group; ^#^ *P* < 0.05 versus TGF-*β*1 group; ^ΔΔ^ *P* < 0.01 versus 5-AZA group (*n* = 3).

The flow cytometric apoptosis assay revealed that the percentage of early apoptotic and late apoptotic/necrotic cells (Figure 3; Q2 + Q4) rate in TGF-*β*1 group had no statistical difference compared with the control group. The percentage of early apoptotic and late apoptotic/necrotic cells rate in the 5-AZA group was significantly higher than that of the control group and the TGF-*β*1 group. The percentage of early apoptotic and late apoptotic/necrotic cells rate in the combined treatment group was significantly lower than in 5-AZA group. However, percentage of early apoptotic and late apoptotic/necrotic cells rate in the combined treatment group showed no statistical difference compared with that of the TGF-*β*1 group or the control group (Figure 3).

### 3.3. Immunofluorescence Staining of Cardiomyocyte-Specific Proteins

To estimate the differentiation level of BMMSCs into cardiomyocyte-like cells, the expression of cardiomyocyte-specific proteins was identified through immunofluorescence staining. Red fluorescence-labeled MHC proteins, CX-43 proteins, green fluorescence-labeled *α*-actin proteins, and cTnI proteins were observed in the cells at the end of the experiment.

**Figure 4:**
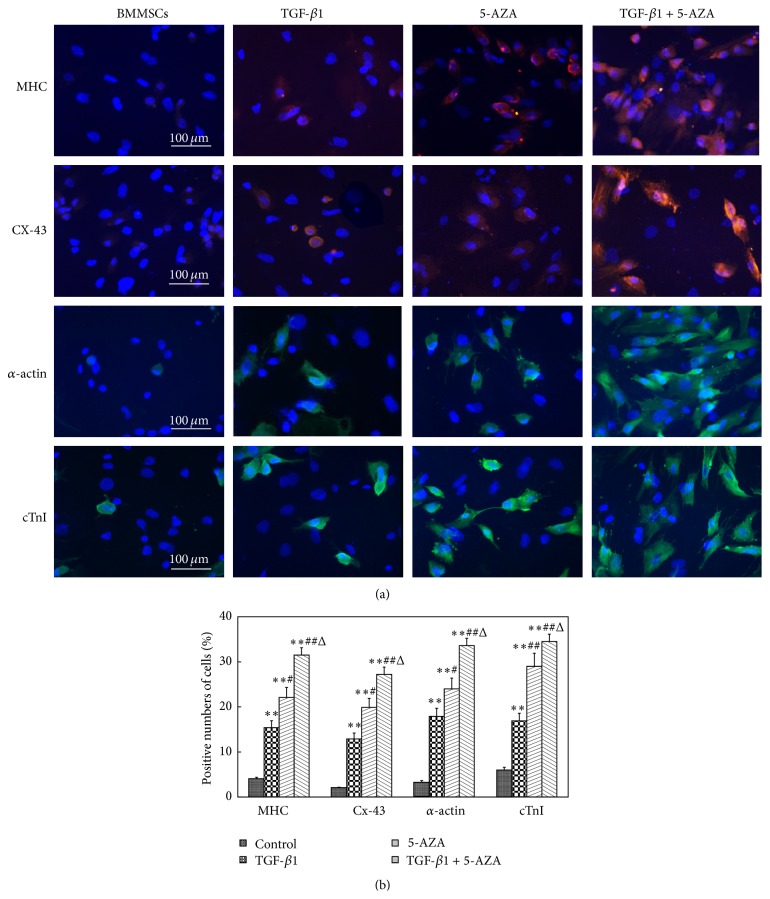
Immunofluorescence staining of cardiomyocyte-specific proteins. (a) The cells positive for MHC, CX-43, *α*-actin, and cTnI were detected by immunofluorescence microscopy (×400). (b) The number of cells positive for MHC, CX-43, *α*-actin, and cTnI. ^*∗∗*^ *P* < 0.01 versus control group; ^#^ *P* < 0.05; ^##^ *P* < 0.01 versus TGF-*β*1 group; ^Δ^ *P* < 0.05 versus 5-AZA group (*n* = 3).

Quantitative analysis revealed that the number of cells positive for MHC, CX-43, *α*-actin, and cTnI in the combined treatment group, TGF-*β*1 + 5-AZA, was noticeably higher than those in the TGF-*β*1 group or the 5-AZA group. The number of cells positive for MHC, CX-43, *α*-actin, and cTnI were higher in the 5-AZA group compared with those in the TGF-*β*1 group (Figures 4(a) and 4(b)).

### 3.4. Differentiation Potential of BMMSCs

**Figure 5:**
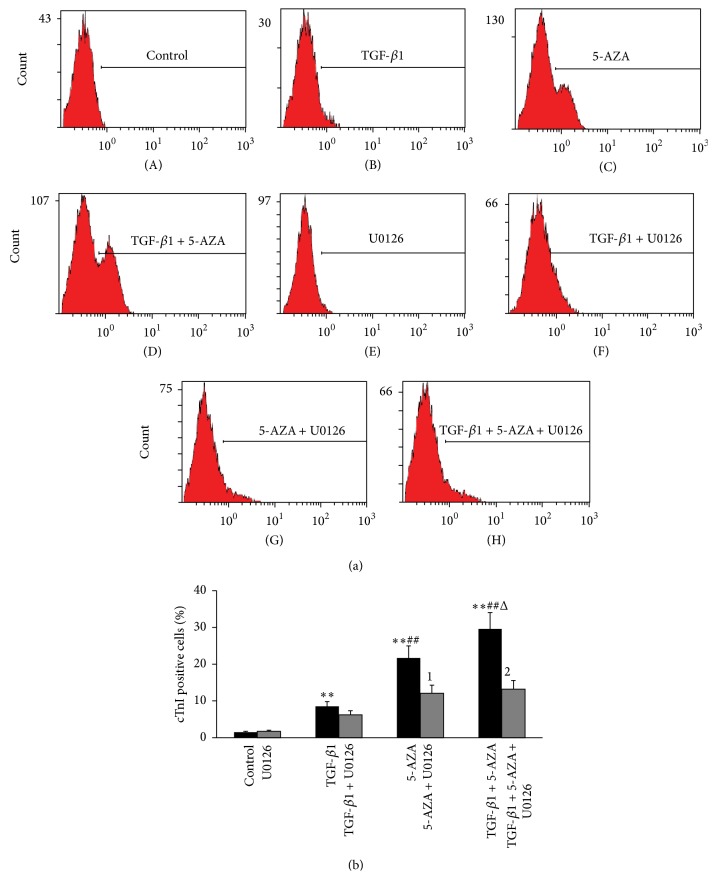
Flow cytometric analysis of differentiation potential of BMMSCs. (a) Graph showing the proportion of positive cells after staining with the cTnI in the different treatment groups and uninduced groups. (b) Histograms showing the proportion of positive cells after staining with the cTnI in treated and uninduced BMMSCs. Results are expressed as the means ± SEM. ^*∗∗*^ *P* < 0.01 versus control group; ^##^ *P* < 0.01 versus TGF-*β*1 group; ^Δ^ *P* < 0.05 versus 5-AZA group; ^1^ *P* < 0.05 versus 5-AZA group; ^2^ *P* < 0.01 versus combined treatment group (*n* = 3).

Flow cytometric experimentation showed that the rates of cTnI positive cells of the combined treatment group, TGF-*β*1 + 5-AZA, were much higher than those of the TGF-*β*1 group (29.6 ± 7.3% versus 8.5 ± 0.9%, *P* < 0.01) or the 5-AZA group (29.6 ± 7.3% versus 21.7 ± 4.4%, *P* < 0.05), alone. The differentiation rate in the 5-AZA group was higher than that of the TGF-*β*1 group (*P* < 0.01). The differentiation rate of the 3 treatment groups was significantly higher than the control group (1.0 ± 0.3%) (*P* < 0.01) (Figures 5(a)(A), 5(a)(B), 5(a)(C), 5(a)(D), and 5(b)). Treatment with U0126 strongly reduced the number of cTnI-positive cells in the 5-AZA group and the combined treatment group (Figures 5(a)(E), 5(a)(F), 5(a)(G), 5(a)(H), and 5(b)).

### 3.5. Protein Expression Levels of cTnI, *α*-Actin, Phosphorylated ErK1/2, and OCT4

**Figure 6:**
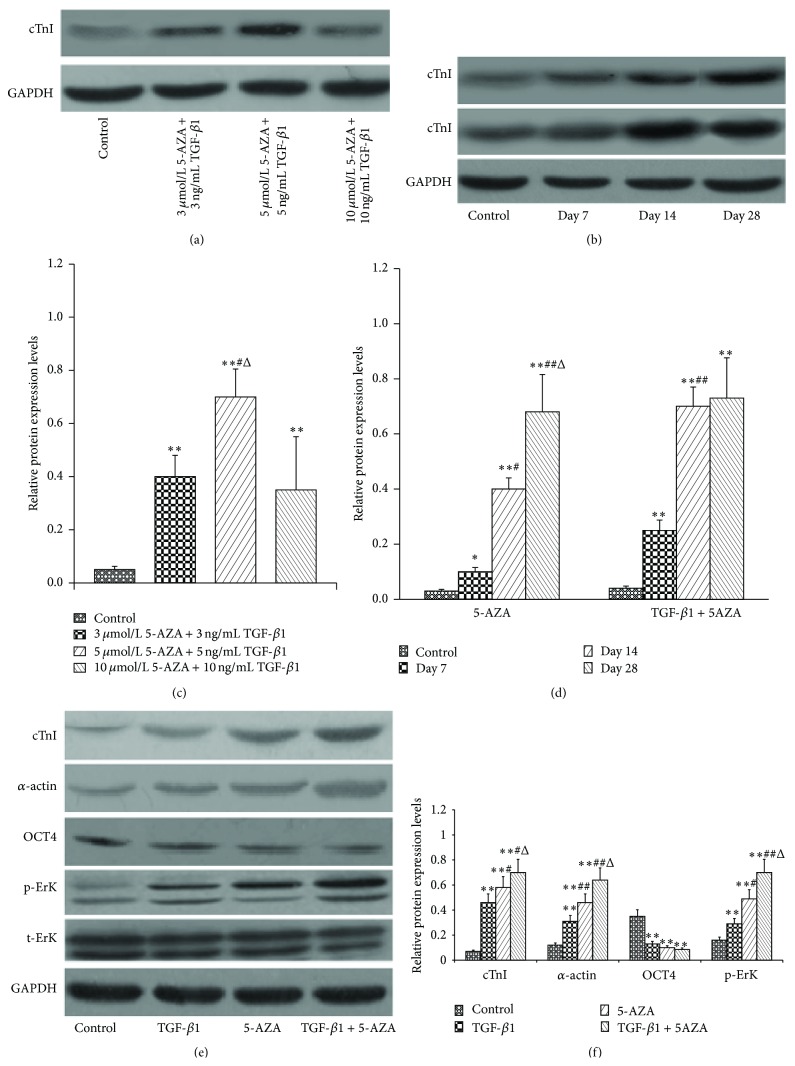
Protein expression levels of cTnI, *α*-actin, p-ErK1/2, and OCT4. (a, c) The protein expression levels of cTnI in the different doses of cocktail. ^*∗∗*^ *P* < 0.01 versus control group; ^#^ *P* < 0.05 versus 3 *μ*mol/L 5-AZA combined with 3 ng/mL TGF-*β*1; ^Δ^ *P* < 0.05 versus 10 *μ*mol/L 5-AZA combined with 10 ng/mL TGF-*β*1. (b, d) The protein expression levels of cTnI at days 0, 7, 14, and 28 in the 5-AZA induction group and in the combination induction group. ^*∗∗*^ *P* < 0.01 versus day 0; ^#^ *P* < 0.05, ^##^ *P* < 0.01 versus day 7; ^Δ^ *P* < 0.05 versus day 14. (e, f) The expression levels of cTnI, *α*-actin, p-ErK1/2, and OCT4 proteins in different treatment groups. ^*∗∗*^ *P* < 0.01 versus control group; ^#^ *P* < 0.05, ^##^ *P* < 0.01 versus TGF-*β*1 group; ^Δ^ *P* < 0.05 versus 5-AZA group (*n* = 3).

In order to determine the optimal dose of cocktail to induce the protein expression of cTnI, we have checked other doses of cocktail, for example, 3 *μ*mol/L 5-AZA combined with 3 ng/mL TGF-*β*1, 10 *μ*mol/L 5-AZA combined with 10 ng/mL TGF-*β*1, to which the protein expression levels of cTnI were inferior to the dose, and 5 *μ*mol/L 5-AZA combined with 5 ng/mL TGF-*β*1, currently in the paper (Figures 6(a) and 6(c)). Our preliminary experimental results showed that using 5-AZA as an inducer, the protein expression levels of cTnI were not in the stable level until 28 days, whereas, in the combination induction group, the protein expression levels of cTnI required only 14 days (Figures 6(b) and 6(d)).

To further verify the differentiation of BMMSCs into cardiomyocyte-like cells, we evaluated the expression levels of cTnI and *α*-actin proteins by western blot analysis. The cTnI and *α*-actin protein expression levels in the 3 experimental groups were significantly higher than in the control group (*P* < 0.01). Moreover, the combined treatment group, TGF-*β*1 + 5-AZA, reveals the strongest expression of 2 cardiac-specific proteins among the 3 treatment groups (*P* < 0.05). The cTnI and *α*-actin protein levels in the 5-AZA group were higher than those in the TGF-*β*1 group (*P* < 0.01) (Figures 6(e) and 6(f)).

To obtain a better understanding of the high differentiation rate of BMMSCs to cardiomyocyte-like cells, we investigated the role of p-ErK1/2, t-ErK, and OCT4. The protein expression level of p-ErK1/2 in the combined treatment group, TGF-*β*1 + 5-AZA, was the highest among the 4 groups. The protein expression level of p-ErK1/2 in the 5-AZA group was higher than that in the TGF-*β*1 group (*P* < 0.01). The OCT4 expression in all the 3 treatments of induced protocols was reduced compared with the control group (*P* < 0.05), but there were no significant differences among the 3 treatment groups (*P* > 0.05) (Figures 6(e) and 6(f)).

## 4. Discussion

The conventional therapeutic modalities for ischemic heart disease have limited success in preventing the progression of left ventricular remodeling and chronic heart failure. Stem cell therapy is fast developing as a promising alternative to current treatments. The therapy of transplanted BMMSCs has been carried out in several animal studies and clinical trials [15–17]. However, insufficient number of cardiomyocytes derived from BMMSCs has become one of the chief obstacles in developing an efficient and effective treatment [6, 18].

Experimental evidence clearly demonstrates that the efficiency of stem cell transplantation may be enhanced to some degree by the directed differentiation of BMMSCs towards a cardiomyogenic lineage* in vitro*, prior to transplantation [18]. Both TGF-*β*1 and 5-AZA are common inducers that are used to induce the differentiation of BMMSCs into cardiomyocyte-like cells* in vitro*. However, 5-AZA can cause an imbalance and affect chromatin organization at the concentration that has an inducing effect, while TGF-*β*1 revealed very low efficiency [10, 11].

In the present study, it was confirmed that BMMSCs could be differentiated into cardiomyocyte-like cells by either TGF-*β*1 or 5-AZA. TGF-*β*1 induced a lower differentiation rate than 5-AZA in BMMSCs, although 5-AZA exhibited toxicity in cell induction. These results are consistent with the findings of the previous study [11, 12]. Furthermore, our data showed that the combined action of low doses of TGF-*β*1 and 5-AZA offers significant advantages in cell proliferation and the differentiation effect of BMMSCs compared with only high dose TGF-*β*1 or high dose 5-AZA. This is evident by the MTS result, the number of cells that stain positive for cardiac-specific markers and the expression of cardiac-specific markers at protein levels. The cardiac-specific markers used in the current study include cardiac contractile proteins (cTnI, *α*-actin, and MHC) and the gap junction protein (CX-43). The combination of these markers represents cardiomyogenic identity very well. The results of the flow cytometric experiments confirmed that the differentiation rate of the combined low dose treatment group, TGF-*β*1 + 5-AZA, was significantly higher than the individual differentiation rate of either high dose TGF-*β*1 or high dose 5-AZA. In addition, the period of induction using the combined low dose treatment of TGF-*β*1 and 5-AZA was shortened to 2 weeks compared to the induction time obtained using only 5-AZA. However, no functional beating cardiomyocytes were observed in all the groups.

The OCT4 is a master transcriptional regulator, which mediates pluripotency in embryonic stem cells (ESCs) and MSCs through inhibition of tissue-specific and promotion of stem cell-specific genes [19, 20]. OCT4 is highly expressed in pluripotent cells and becomes downregulated with loss of pluripotency [21, 22]. The current results demonstrate that pluripotency is reduced in cardiomyocyte-like cells derived from BMMSCs. To identify the signaling pathways involved in inducing BMMSCs, we examined the status of p-ErK1/2 after treatment. Our data revealed that both TGF-*β*1 and 5-AZA induced ErK phosphorylation in BMMSCs. The level of p-ErK1/2 in the combined low dose treatment group, TGF-*β*1 + 5-AZA, was higher than that of either high dose TGF-*β*1 or high dose 5-AZA. Experimental evidence revealed that 5-AZA induced ErK phosphorylation in human umbilical cord-derived MSCs and ESCs, while U0126, the ErK inhibitor, significantly inhibited the expression of cardiac-specific genes and proteins induced by 5-AZA [23–25]. Fukuda reported that p-ErK1/2 increased in cells from murine BMMSCs after 5-AZA exposure [25]. Besides, TGF-*β*1 induces an increase in p-ErK1/2 in the 10T1/2 multipotent mesenchymal cell line [26]. Our data suggest that the mechanism of upregulated cardiac differentiation by the combined low dose treatment of TGF-*β*1 and 5-AZA was associated with the crosstalk of these 2 inducers at the level of p-ErK1/2. However, the specific mechanism of these processes still needs to be further investigated.

## 5. Conclusion

The present study demonstrates that combined low dose treatment of TGF-*β*1 and 5-AZA offers significant advantages in cell viability and differentiation of BMMSCs. The possible mechanism underlying these effects may involve p-ErK1/2. The combined low dose treatment of TGF-*β*1 and 5-AZA could be a safe and effective method to induce differentiation. Future studies should analyze the mechanism underlying the combined induction effect of these two inducers and the potential applications* in vivo*.
